# Assessment of biotinidase activity changes over time in biotinidase deficient patients

**DOI:** 10.3389/fped.2026.1694031

**Published:** 2026-03-02

**Authors:** Aliye Gülbahçe, Ahmet Muderrisoglu, Hatice Güneş, Murat Erdoğan, Munis Dündar, Fatih Kardaş

**Affiliations:** 1 Pediatric Nutrition and Metabolism Clinic, Kocaeli City Hospital, Kocaeli, Türkiye; 2 Department of Pediatric Nutrition and Metabolism, Faculty of Medicine, Erciyes University, Kayseri, Türkiye; 3 Department of Pharmacology, Faculty of Medicine, Kırıkkale University, Kırıkkale, Türkiye; 4 Clinic of Genetics, Kayseri City Hospital, Kayseri, Türkiye; 5 Department of Medical Genetics, Faculty of Medicine, Erciyes University, Kayseri, Türkiye

**Keywords:** biotin, biotinidase deficiency, *BTD* gene, consanguinity, pathogenic variants

## Abstract

**Introduction:**

Biotinidase enzyme is responsible for recycling biotin which is essential for metabolic functions. Loss of function mutations in the *BTD* gene causes biotinidase deficiency (BTD). It is diagnosed by measuring biotinidase activity and it can lead to severe neurological symptoms. We aimed to evaluate biotinidase activity changes in patients with BTD over time.

**Methods:**

194 patients with BTD were enrolled. Clinical, laboratory and genetic data of the patients were retrospectively evaluated. Patients with enzyme activity below 10% of normal were diagnosed with profound BTD while patients with enzyme activity between 10% and 30% were diagnosed with partial BTD.

**Results:**

104 (53.6%) patients were male, most patients were diagnosed at screening (*n* = 183, 94.3%) and the mean age at the time of diagnosis for symptomatic patients was 82.7 ± 22.8 (range: 1–216) months. Two (1%) patients had profound BTD, 168 (86.6%) patients had partial BTD, and 24 (12.4%) patients had more than 30% of normal biotinidase activity. Overall, the last measured biotinidase activity levels were significantly higher than the initial measurements (*p* < 0.0001). This finding was valid for all subgroups classified according to birth week, birth weight, and consanguineous marriage status. The increase in enzyme rate over time was slower in children of consanguineous marriages compared to children who were not.

**Discussion:**

This study showed that biotinidase activity increased in BTD patients over time and repeated measurements of biotinidase would be a better approach to evaluate BTD. In addition, consanguineous marriage may be a risk factor for a worse prognosis in BTD.

## Introduction

Biotin (vitamin B_7_) plays a crucial role in a wide range of metabolic processes such as utilization of carbohydrates, fats, and amino acids by acting as a coenzyme for carboxylase enzymes. No endogenous free biotin synthesis occurs and the need for biotin is supplied from biotin cycle that recovers free biotin taken from food. Without normal biotinidase function, biotin cycle becomes impaired (1, 2).

Insufficient levels of free biotin are the result of biotinidase deficiency (BTD). BTD (OMIM#253260) is caused by loss of function mutations in the *BTD* gene that is responsible for coding biotinidase (3). There are several frameshift, deletion or missense variants identified in the *BTD* gene found to be associated with loss of biotinidase function. Among them, *BTD* c.1330G>C, c.470G>A and, c.1368A>C pathogenic variants are the most common (4). Highest reduction in biotinidase function occurs mostly when two or more different loss of function mutations are present in the *BTD* gene (compound mutation carrier) (4, 5). BTD is inherited in an autosomal recessive manner and its frequency is higher in countries that have a population with a high rate of consanguinity (6). Biotinidase activity level is the main factor that determines the course of the disease so, BTD is classified according to the activity level of biotinidase in the serum. While lower than 10% of normal biotinidase activity is defined as profound BTD, activity levels between 10% and 30% are classified as partial BTD (7). BTD can be diagnosed during newborn screening when symptoms do not yet develop or in untreated individuals after the development of symptoms (8). Symptoms only occur in untreated patients and some of them are seizures, optic atrophy, hearing loss, developmental delay, skin rash, and respiratory problems (8).

Both types of BTD are treated with biotin supplementation. Most symptoms resolve with the initiation of biotin. However, neurological symptoms such as optic atrophy are not fully resolvable (9). In addition, the measurement of serum biotinidase activity is the main parameter that is checked during patient check-ups to assess the state of the disease because it is the main factor that determines the severity of the disease (8). There is no consensus for the initiation of biotin in BTD patients diagnosed with genetic testing but have higher than 30% of normal serum biotinidase activity (10).

One of the most important aspects of BTD management is to prevent the development or progression of neurological symptoms because some of these symptoms are the result of neurological damage that is irreversible in nature. Because of this, adherence to the biotin treatment, which is essential for preventing neurological symptom development, is the most important part of managing BTD (8). Low treatment adherence can result in the development of neurological symptoms which can lead to disability (8). Therefore, the necessity for lifelong therapy which reduces patient compliance is a discomforting aspect of BTD surveillance (11).

Understanding the course of biotinidase activity level over time is also important for BTD management because most clinicians prefer to manage their patients' treatment according to the biotinidase activity level. There are some studies in the literature indicating that biotinidase activity levels may elevate over time (10, 12). This finding has the potential to change clinicians' approach to conducting BTD treatment. Therefore, it is important to reach a conclusion on whether biotinidase activity elevates over time and if so, how this change should affect BTD management.

In this study, we aimed to evaluate the changes in biotinidase activity over time in BTD patients to contribute to the knowledge of BTD management.

## Materials and methods

All procedures performed in studies involving human participants were in accordance with the ethical standards of the institutional and/or national research committee and with the 1964 Helsinki Declaration and its later amendments or comparable ethical standards. The study was approved by the Local Ethics Committee of the Erciyes University (No: 2023/609).

Patients were recruited from the pediatric nutrition and metabolism clinic of Erciyes University Hospital. BTD was diagnosed either by screening (national newborn screening program or family screening) or by biotinidase activity measurement after manifestation of possible symptoms. Definitive BTD diagnosis was made after multiple measurements of serum biotinidase activity. Participants were separated into 3 groups according to their biotinidase activity level; profound deficiency, partial deficiency, and normal activity. While profoundly and partially biotinidase deficient patients received biotin supplementation as a treatment, participants in the normal activity group have not received biotin. Participants with normal biotinidase activity were included into the study to investigate suspected increase in biotinidase activity rate over time among all activity groups and whether biotin supplementation has any effect on enzyme activity rate. Demographics, results of the laboratory tests (biotinidase activity level, analyses of urine organic acids, and carnitine), *BTD* gene sequencing, physical examination, screening, and hearing test were retrospectively analyzed. We also evaluated the measurements of biotinidase activity at four different times. Mean time at first, second, third, and fourth measurements were 6.7, 18.7, 29.6, and 37.9 months, respectively.

Serum biotinidase activity levels were measured quantitatively by the colorimetric method. Cases with biotinidase activity below 1.05 U/L, calculated as under 10% of normal activity, are considered profound, and cases with values between 10% and 30%, calculated as 1.05–3.15 U/L, are considered partial biotinidase deficiency (13).

For genetic analysis, DNA was isolated from 200 µL of venous blood using the EZ1 Advanced Automated Solutions (Qiagen, Hilden, Germany). Polymerase chain reaction (PCR) was used to amplify the *BTD* gene (NM_001281724.2). Used PCR primers were provided in Supplementary Table S1. The National Center for Biotechnology Information database (https://www.ncbi.nlm.nih.gov) was used to check PCR product sequence and designing PCR primers. Sanger sequencing, using the 3,500 Series Genetic Analyzers (Thermo Fischer Scientific, Waltham, Massachusetts, USA) and SeqScap Software v3.0 (Applied Biosystems, Waltham, Massachusetts, USA), was used to analyze PCR products. Pathogenic variants were evaluated according to the American College of Medical Genetics and Genomics classification. We also checked the ClinVar database (https://www.ncbi.nlm.nih.gov) for assessing variant frequency and pathogenicity.

Statistical analyses were performed by using GraphPad Prism version 8 (GraphPad Software, California, USA). Descriptive statistics were shown as mean ± standard deviation (95% confidence intervals) for the results of the parametric tests and, median [interquartile range (IQR)_25_–IQR_75_] for the results of the non-parametric tests. Shapiro–Wilk test was used to assess the normality distribution of the numerical data. t-test, one-way ANOVA, and *post-hoc* Tukey's tests were used for the data that were normally distributed while, Mann–Whitney U, Kruskal–Wallis, and *post-hoc* Dunn's tests were used for the data that were not normally distributed. In addition, paired t and Wilcoxon tests were performed for the comparison of paired data, where applicable. Pearson correlation analysis was also performed for evaluating the association of enzyme activity with time. Genetic data was evaluated by using the SNPStats web analyzing tool (snpstats.net/start.htm) and the chi-square test. *p* < 0.05 was accepted as statistically significant.

When we used the values found in our study (difference in population means: 0.55, standard deviation of difference in the response of matched pairs: 1, and type I error probability: 0.05) for statistical power calculation, we found the statistical power of Wilcoxon test comparing overall initial and last measured biotinidase activities to be higher than 0.8.

## Results

A total of 194 patients' retrospective data were analyzed, and the median age was 51 months (min: 2, max: 252). Demographics and clinical findings are shown in Table 1. Median age at diagnosis for symptomatic patients was 64 (1–216) months.

**Table 1:** Demographic and clinical characteristics of patients.

Subgroups	*n* (%)
Clinical data (*n* = 194)	
Male/Female	104 (53.6)/ 90 (46.4)
Consanguineous Marriage	47 (24.2)
Partial Deficiency	41 (87.2%)
Normal Enzyme Activity	6 (12.8%)
Birth Week	
Premature Birth	28 (19.4)
Mature Birth	116 (59.8)
N/A	50 (20.8)
Birth Weight	
SGA	21 (10.8)
AGA	111 (57.2)
LGA	5 (2.6)
N/A	57 (29.4)
Diagnosed at (*n* = 194)	
National Newborn Screening	175 (90.2)
Family Screening	8 (4.1)
Biotinidase activity measurement after manifestation of symptoms	11 (5.7)
Diagnosis (*n* = 194)	
Profound Deficiency	2 (1)
Partial Deficiency	168 (86.6)
Normal Enzyme Activity	24 (12.4)
Clinical Manifestation of Symptomatic Patients (*n* = 11)	
Ataxia	1 (9.1)
Attention Deficit + Hearing Loss	1 (9.1)
Cardiomyopathy	1 (9.1)
Dermatological Findings	1 (9.1)
Epilepsy	4 (36.3)
Left Ventricular Hypertrophy	1 (9.1)
Respiratory Distress	1 (9.1)
Optic Atrophy	1 (9.1)

SGA, small for gestational age; AGA, appropriate for gestational age; LGA, large for gestational age; N/A, not available.

Pearson correlation analysis, which evaluated the association between biotinidase activity rate and time, showed positive correlation between enzyme activity level and time (months) in both patients with partial BTD (*r*^2^ = 0.084, *p* < 0.0001) and participants with normal biotinidase activity (*r*^2^ = 0.023, *p* = 0.017). Neither biotinidase activity at diagnosis nor the last measured biotinidase activity was different between the genders. Biotinidase activity levels at diagnosis between patients with partial BTD and patients with normal biotinidase activity (higher than 30%) as well as patients with profound BTD and patients with normal biotinidase activity were found to be significantly different as expected (*p* < 0.0001 for both comparisons). In addition, the last measured biotinidase activity levels were only significantly different between profoundly deficient patients and patients who have normal biotinidase activity (adjusted *p* = 0.017). Comparisons of biotinidase activity levels between the groups according to birth week (premature vs. mature) and birth weight [small for gestational age (SGA) vs. appropriate for gestational age (AGA) vs. large for gestational age (LGA)] yielded no statistically significant results expect from the comparison of last measured biotinidase activities of groups according to birth weight. Unexpectedly, the last measured biotinidase activity of patients with SGA was higher than patients with AGA (adjusted *p* = 0.036). While the rate of partial deficiency and level of biotinidase activity at diagnosis was similar between the groups according to consanguineous marriage status, the last measured biotinidase activity level was significantly different between the groups (*p* = 0.0003). Results are presented in Table 2.

**Table 2:** Comparisons of initial and last biotinidase activities between the groups.

Subgroups	Biotinidase Activity Level at Diagnosis (U/L)	Last Measured Biotinidase Activity Level (U/L)
Gender		
Male	2.8 (2.2–3)	3 (2.3–3.4)
Female	2.8 (2.3–3)	3.1 (2.6–3.6)
***p* Value**	0.49	0.1
Diagnosis		
Profound Biotinidase Deficiency	1 (1–1)	1.6 (1.2–2)
Partial Biotinidase Deficiency	2.7 (2.2–2.9)	3 (2.5–3.5)
Normal Biotinidase Activity	3.3 (3.1–3.5)	3.2 (3.1–3.7)
***p* Value**	**<0.0001**	**0.007**
Birth Week		
Premature Birth	2.9 (2.6–3)	3.1 ± 0.8 (2.8–3.5)
Mature Birth	2.8 (2.5–3)	3.1 ± 0.7 (3–3.2)
***p* Value**	0.962	0.76
Birth Weight		
SGA	2.8 (2.4–2.9)	3.5 ± 0.8 (3.1–3.8)
AGA	2.9 (2.5–3)	3.1 ± 0.7 (2.9–3.2)
LGA	2.9 (1.2–3)	2.9 ± 0.8 (0.9–5)
***p* Value**	0.423	**0.043**
Consanguineous Marriage		
Yes	2.6 ± 0.6 (2.5–2.8)	2.9 (2–3.3)
No	2.7 ± 0.5 (2.6–2.8)	3.1 (2.8–3.7)
***p* Value**	0.289	**0.0003**

SGA, small for gestational age; AGA, appropriate for gestational age; LGA, large for gestational age.

The results of the parametric tests were shown as mean ± standard deviation (95% confidence intervals) while the results of the non-parametric tests were demonstrated as median (IQR_25_–IQR_75_).

Statistically significant *p* values were marked as bold.

**Figure 1:**
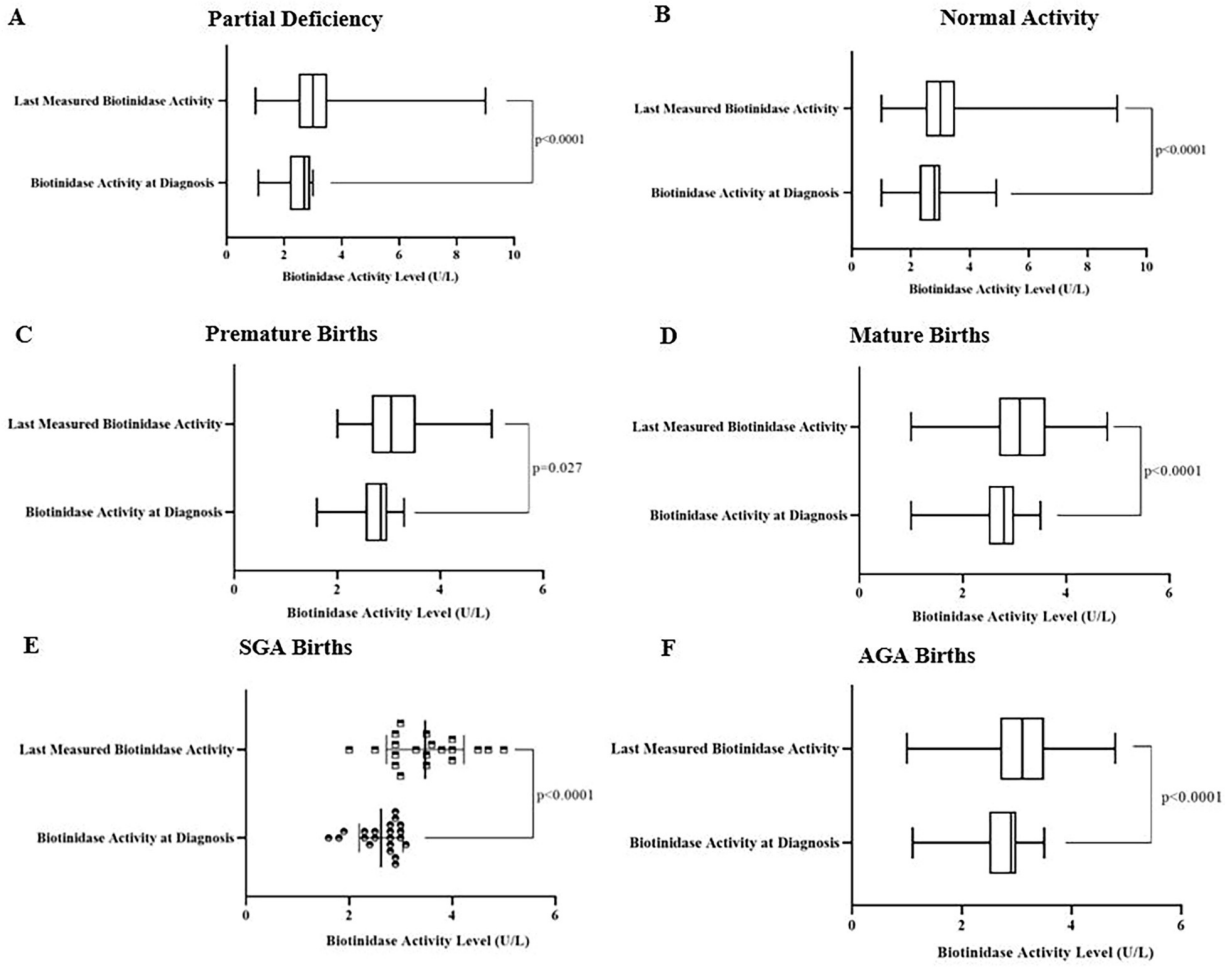
Initial and last biotinidase levels were 2.7 (2.2–2.9) U/L and 3 (2.5–3.5) U/L for patients with partial BTD, respectively (*p* < 0.0001) **(A)**. Initial and last biotinidase levels in participants with normal biotinidase activity were 2.9 (2.7–3) U/L and 3.5 (3.2–3.9) U/L (*p* < 0.0001) **(B)**. Diagnosis and last measured biotinidase activity levels were 2.85 (2.55–2.98) U/L and 3.05 (2.68–3.53) U/L in patients with premature birth, respectively (*p* = 0.027) **(C)**. Last measured biotinidase activities [3.1 (2.7–3.6) U/L] were significantly higher than biotinidase activity levels at diagnosis [2.8 (2.5–3) U/L] in patients with mature births (*p* < 0.0001) **(D)**. Significantly elevated last measured biotinidase activities compared to biotinidase activity levels at diagnosis were found in patients with SGA births [3.48 ± 0.75 (3.12–.83) U/L vs. 2.62 ± 0.43 (2.42–2.81) U/L, *p* < 0.0001] **(E)**. Last measured biotinidase activities were significantly increased compared to biotinidase activity levels at diagnosis in patients with AGA births [3.1 (2.7–3.5) U/L vs. 2.9 (2.5–3) U/L, *p* < 0.0001] **(F)**. Results of the parametric and non-parametric tests were presented as mean ± standard deviation (95% confidence intervals) and median (IQR_25_–IQR_75_), respectively.

Last measured enzyme activities were significantly higher than enzyme activities at diagnosis in patients with partial BTD (*p* < 0.0001). The same result was valid for participants with normal biotinidase activity (*p* < 0.0001). Moreover, significantly higher last measured biotinidase activity levels compared to levels at diagnosis were found in patients with premature birth (*p* = 0.027), patients with mature birth (*p* < 0.0001), patients with SGA (*p* < 0.0001), and patients with AGA (*p* < 0.0001). Results are demonstrated in Figure 1. Biotinidase activities of 100% of profoundly deficient patients, 71.6% of partially deficient patients, 50% of patients with normal enzyme activity, and overall, 69.9% of all patients were found to be elevated over time.

**Figure 2:**
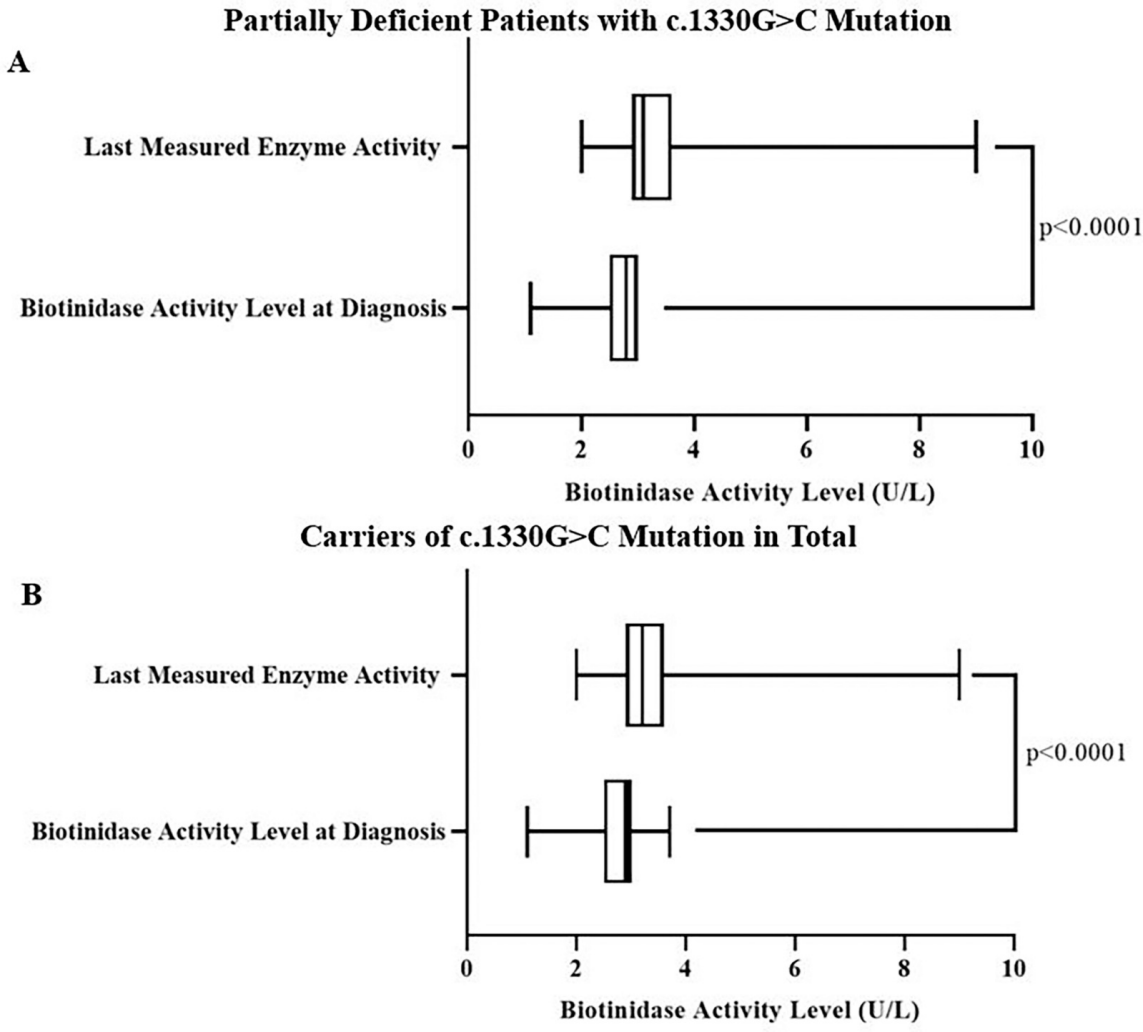
Last measured biotinidase activities of carriers for the *BTD* c.1330G>C variant was significantly higher than the levels at diagnosis in partially biotinidase deficient patients **(A)** as well as in total **(B)** (3.1 (2.9–3.6) U/L vs. 2.8 (2.5–3) U/L and 3.2 (2.9–3.6) U/L vs. 2.9 (2.5–3) U/L, respectively, *p* < 0.0001 for both comparisons).

**Figure 3:**
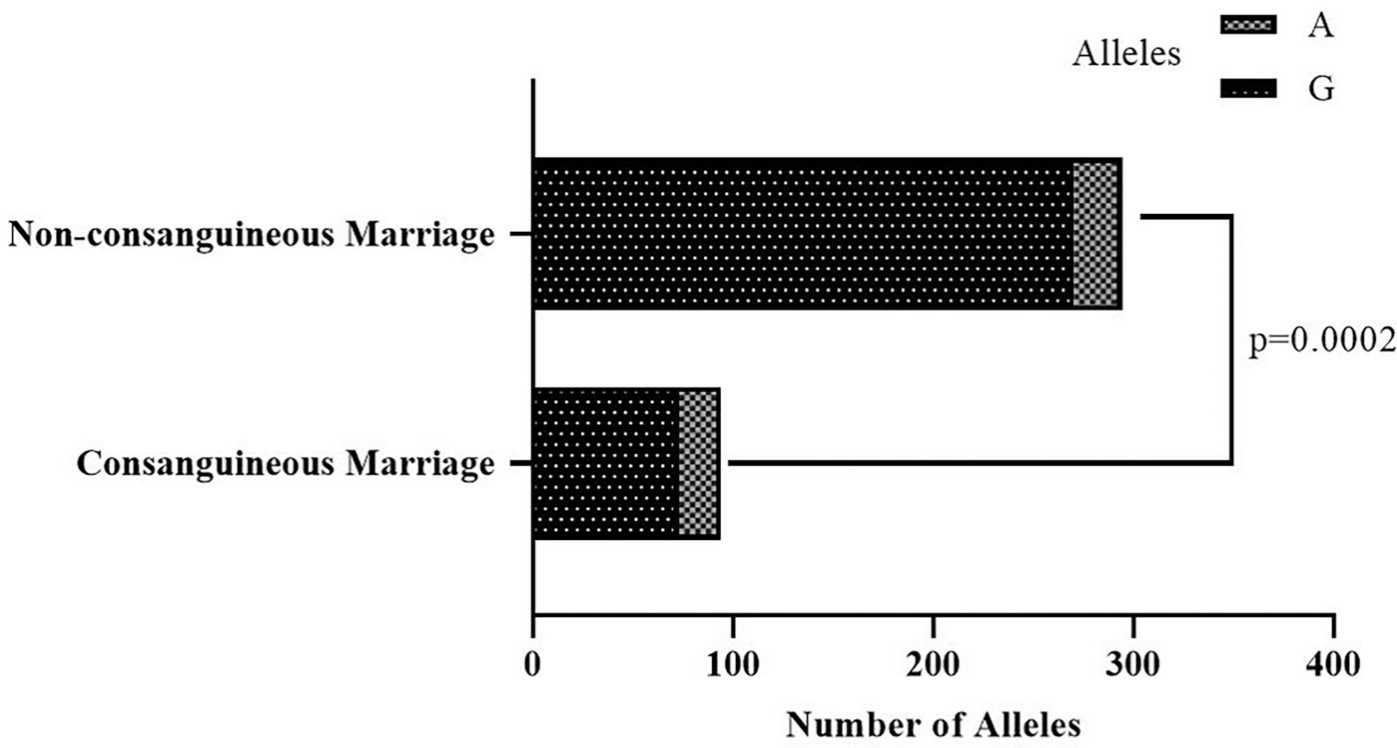
Frequency of the variant A allele for the *BTD* c.470G>A variant was significantly higher in children of consanguineous marriage than the children of non-consanguineous marriage (22.3% vs. 8.2%, *p* = 0.0002).

Overall variant allele frequencies for the examined genetic variants in the *BTD* gene are shown in Supplementary Figure S1. The two of the most common variants were c.1330G>C (31.2%) and c.470G>A (14.4%). The last measured biotinidase activities of carriers for the *BTD* c.1330G>C variant was significantly higher compared to the levels at diagnosis in patients with partial BTD as well as in total (*p* < 0.0001 for both comparisons) (Figure 2). On the other hand, initial and last enzyme activities among *BTD* c.470G>A variant carriers and non-carriers were similar. We also found no difference between partially biotinidase deficient patients and patients with normal biotinidase activity regarding the frequency of the variant allele for the *BTD* c.1330G>C and c.470G>A variants. However, the rate of the variant A allele for the *BTD* c.470G>A mutation was significantly higher in children of consanguineous marriage than the children of non-consanguineous marriage (*p* = 0.0002, Figure 3). Analysis of the other genetic variants yielded no statistically significant results. The results of the genetic analysis are presented in Supplementary Table S2.

**Figure 4:**
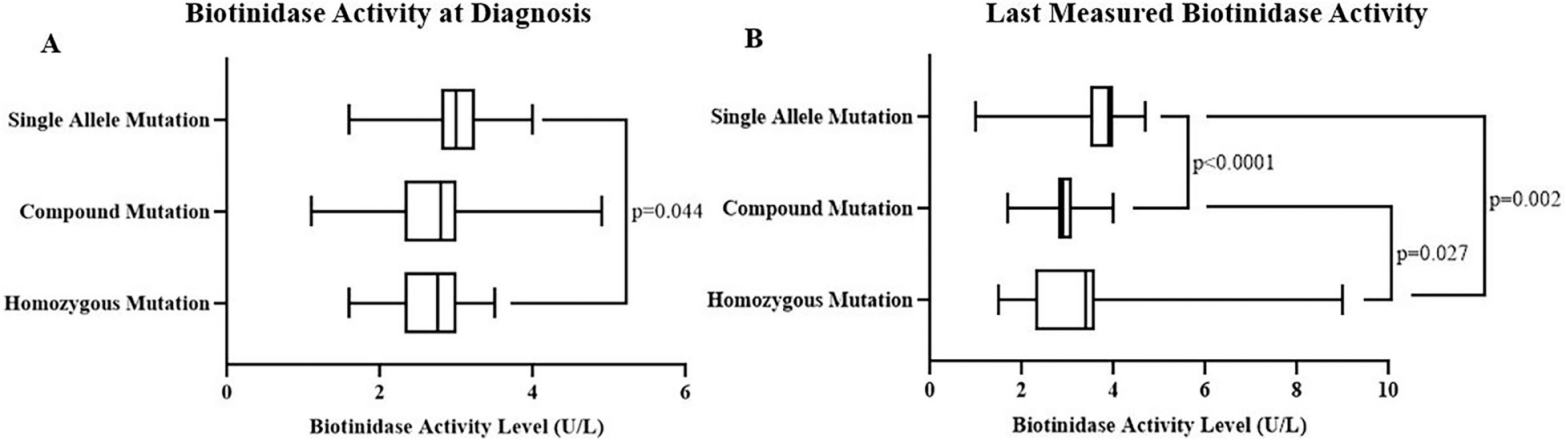
Initial biotinidase activity level in homozygous mutation carriers [2.75 (2.33–3) U/L] was significantly lower than the level in single mutation carriers [3 (2.8–3.25) U/L] (adjusted *p* = 0.044) **(A)**. The last measured biotinidase activities were 3.4 (2.3–3.6) U/L, 2.9 (2.78–3.1) U/L, and 3.9 (3.5–4) U/L for homozygous, compound, and single mutation carriers, respectively (*p* < 0.0001) **(B)**.

**Figure 5:**
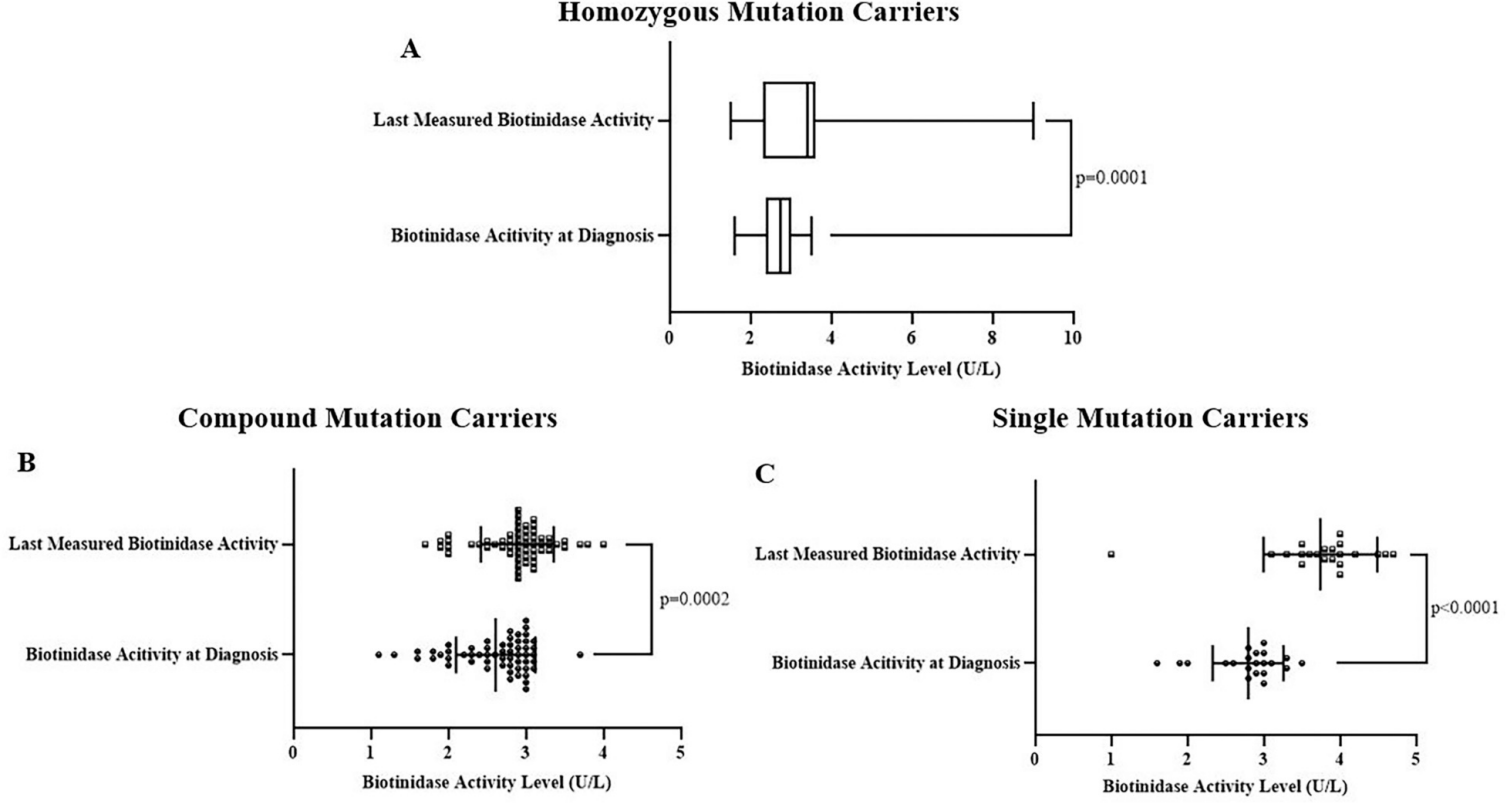
The last measured biotinidase activity [3.4 (2.3–3.6) U/L] was significantly higher compared to initial activity [2.75 (2.38–3) U/L] in homozygous mutation carriers (*p* = 0.0001) **(A)**. Last [2.88 ± 0.47 (2.76–3) U/L] and initial [2.61 ± 0.51 (2.48–2.75) U/L] biotinidase activities differed significantly in compound mutation carriers (*p* = 0.0002) **(B)** as well as in single mutation carriers [3.74 ± 0.75 (3.4–4.1) U/L vs. 2.8 ± 0.47 (2.58–3) U/L, *p* < 0.0001] **(C)**.

We also performed genetic analysis between the groups classified as single mutation carriers (single variant allele in one of the examined variants), compound mutation carriers (single variant allele in multiple examined variants), and homozygous mutation carriers (variant genotype in one or more of the examined variants). There was no difference between these groups regarding the type of BTD diagnosis (Supplementary Table S3). However, diagnosis and last measured biotinidase activities were significantly different among these groups (*p* values were 0.033 and <0.0001, respectively, Figure 4). Further, in-group comparisons showed that the last measured biotinidase activity level was significantly higher than the biotinidase activity level at diagnosis in homozygous (*p* = 0.0001), compound (*p* = 0.0002), and single mutation carriers (*p* < 0.0001) (Figure 5).

Levels of urine organic acids were normal in all patients and carnitine level was found to be elevated in only one partially deficient patient.

The cases that caught our attention in the clinical evaluation were as follows:

Despite showing a normal level of biotinidase activity in the newborn screening, a patient was diagnosed with partial BTD after serum biotinidase activity measurement following an observation of a dermatological finding in the physical examination. The patient was 64 months old at the time of diagnosis and found to be a carrier for the *BTD* c.470G>A variant.

Patients with an irreversible neurological finding (one had hearing loss and the other had optic atrophy) were carriers of the *BTD* c.1330 G>C genetic variant. In addition, the patient with optic atrophy had *BTD* c.98_104del7ins3 variant. All reversible symptoms were improved after the initiation of biotin but these two irreversible symptoms were permanent.

## Discussion

The main findings of our study were that overall biotinidase activity rate increased over time, the rate of increase was noticeably slower in patients whose parents are in a consanguineous marriage, and noticeably faster in *BTD* c.1330 G>C genetic variant carriers. Our study also showed different enzyme rates among groups divided according to birth weight and pathogenic variant carrier status.

It is well known that consanguineous marriage is a risk factor for BTD (8). A higher rate of BTD incidence compared to global average was reported for Turkey (14). In addition, several Turkish studies reported 29.1% to 61.5% of consanguinity between parents in families with a BTD child (15, 16). We found a lower than previously reported rate of patients whose parents are in a consanguineous marriage in our study (24.2%). This could be a result of increasing public knowledge regarding the risks of consanguineous marriage on children. In fact, the Turkish Statistical Institute reported that the percentage of consanguineous marriage in new weddings decreased from 5.9% in 2010 to 3.2% in 2023 (17).

There was no difference between the patients whose parents are in a consanguineous marriage and patients whose parents are not regarding the biotinidase activity level at diagnosis. However, the last measured biotinidase activity level among these two groups was different. This has resulted from 4.6% vs. 22.2% mean rate of enzyme activity increase in children of consanguineous marriage and children who are not, respectively. While our study showed overall biotinidase activity elevation over time, the rate of enzyme activity increase in patients whose parents are in a consanguineous marriage was relatively slower. This finding can be a result of a higher frequency of variant allele for the *BTD* c.470G>A mutation in children of consanguineous marriage compared to children who are not (Figure 3). However, our finding of similar levels of biotinidase activities among carriers and non-carriers of variant allele for the *BTD* c.470G>A mutation indicates that a slower increase in enzyme activity rate in these patients might be due to yet-to-be-discovered pathogenic variants affecting biotinidase activity rate since children of consanguineous marriage are well known to be under increased risk for pathogenic variants. Moreover, slow enzyme activity recovery rate in children of consanguineous marriage may lead to worse BTD prognosis since main factor that determines the course of BTD is known to be biotinidase activity rate (7).

Biotinidase activity increased in both BTD patients and participants with normal biotinidase activity as age increases and, we observed no effect of initial biotinidase activity level, birth week, and birth weight on the rate of biotinidase activity increase as levels of biotinidase activity increased across all groups divided according to BTD type, birth week, and birth weight similarly (Table 2, Figure 1). This finding is consistent with the results of several previous studies. Forny et al. showed recovery of enzyme activity with increasing age and Kara et al. found decreasing numbers of profoundly deficient patients when enzyme activity measurements were repeated over time (10, 12). Biotin's impact on the expression of genes responsible for coding biotin-dependent carboxylases by affecting intracellular cGMP, and reduced availability of biotin leading to reduced expression holocarboxylases synthetase have been shown (1, 18). Therapeutic doses of biotin supplementation were also indicated as the cause of the enzyme activity increase with age by acting on gene expression (12). However, our study did not support this assumption as enzyme activity level increased in both biotinidase deficient patients who were receiving biotin supplementation and participants with normal biotinidase activity who did not receive biotin. On the other hand, Forny et al. suggest that their finding of recovery of enzyme activity with age would impact BTD treatment regimens as they recommend a reassessment of biotinidase activity at the age of 5 in BTD patients and stopping biotin supplementation if biotinidase activity exceeds 30% together with no occurrence of symptoms (12). We would like to note that there may be a need for reclassifying patients according to enzyme activity level after some time. Moreover, we noticed a decrease in some patients' enzyme activity levels over time and reclassification would benefit these patients.

Biotinidase activity increased in all groups according to birth week and weight (Figure 1) but it was most prominent in patients with SGA births. It has been shown that impaired biotinidase activity due to liver immaturity recovers over time in prematures (7). Liver immaturity was also observed in SGA births and it was reported that SGA births are more susceptible to metabolic imbalances than AGA births (19). Initial biotinidase activity level of SGA births was slightly but not significantly lower than AGA and LGA births. Yet, SGA births' enzyme activity level significantly exceeded others with advancing age. This finding indicates that it may be insufficient to diagnose BTD with one biotinidase activity measurement. Therefore, we suggest evaluating BTD with repeated biotinidase activity measurements. The same approach was previously recommended by Kara et al. (10) considering their observation of enzyme activity increase in patients with *BTD* c.1330G>C variant (10).

Similar to the results of some previous studies, the most frequent *BTD* gene variant found in our study was c.1330G>C mutation (Supplementary Figure S1) (20). This variant has been shown to reduce biotinidase activity by ∼50% and is associated with partial BTD (3). Swango et al. indicated that partial BTD usually develops when the c.1330G>C variant is in combination with another variant that causes loss of biotinidase function (5). Since BTD diagnosis requires enzyme activity levels equal to or under 30% and c.1330G>C variant is not capable of reducing enzyme activity that much on its own, the authors concluded that further studies are needed to determine the necessity for biotin supplementation in carriers of only *BTD* c.1330G>C variant (5). Later studies showed c.1330G>C variant's association with various symptoms such as seborrheic dermatitis and autism spectrum disorder (21). With this newer information, most clinicians prefer to initiate biotin supplementation to carriers of only *BTD* c.1330G>C variant even if they do not have lower than 30% enzyme activity. As can be expected from the mentioned reasons above, single variant allele carriers for this variant in our study were either partially deficient or had normal levels of enzyme activity. We also found no difference between partially deficient patients and patients with normal enzyme activity regarding the variant allele frequency for the *BTD* c.1330G>C variant (Supplementary Table S2). This result was expected as this variant has been shown to be associated with higher than 30% enzyme activity in addition to lower than 30% enzyme activity (5). On the other hand, both Forny et al. and Kara et al. reported that enzyme rate increase with age was associated with the *BTD* c.1330G>C variant (10, 12). We made the same observation as enzyme activity significantly increased in patients with c.1330G>C variant in our study (Figure 2).

The most concerning aspect of BTD is the occurrence of irreversible neurological symptoms (8). The most common variant of *BTD* was found to be c.98_104del7ins3 variant in BTD patients with a neurological symptom in a study by Karaca et al (22). There were two patients with an irreversible neurological symptom in this study (Table 1). The one patient with optic atrophy had two pathogenic variants; c.98_104del7ins3 and c.1330G>C. Together with the results of the mentioned studies, this finding suggests that BTD patients with c.98_104del7ins3 variant carriers are at a higher risk for developing neurological symptoms and biotin therapy should be initiated in these patients regardless of the measured enzyme activity level.

Initial biotinidase activity level was higher in single mutation carriers than homozygous mutation carriers and the last measured biotinidase activity level was higher in single mutation carriers than both homozygous and compound mutation carriers regardless of BTD diagnosis (Supplementary Table S3, Figure 4). This result can be due to the expected reduction in biotinidase activity level with the increasing number of loss of function mutations (20). In addition, the increase in biotinidase activity over time was observed in all groups classified according to pathogenic variant carrying status (Figure 5). It came to our attention that the last enzyme activity level in compound mutation carriers was lower compared to homozygous mutation carriers and enzyme activity elevation was the lowest in compound mutation carriers (Mean ratio of enzyme activity increases were 21.4%, 8.7%, and 27.8% for homozygous, compound, and single mutation carriers, respectively). These findings suggest that enzyme activity is most impaired when variant alleles are in different locations in the BTD gene rather than when the variants are in the same location. Indeed, a similar interpretation can be made for the results of various previous studies (5, 10, 20).

There were limitations in our study. In our clinic, the initial enzyme activity measurement was planned for the first month of life following newborn screening. Subsequent measurements were scheduled between 6 and 9 months, 12 and 24 months, and 3 and 5 years of age. However, during our retrospective evaluation, we were unable to obtain measurement results in this time table, which prevented the standardization of enzyme analysis measurements timing. In addition, our claim that consanguinity may be an indicator for worse prognosis is based upon biotinidase activity measurements and, lacks support from clinical data. Because most patients were diagnosed before manifestation of any symptoms, we were unable to compare the clinical course of BTD between children of consanguineous marriage and other children.

In conclusion, the results of this study indicate that biotinidase activity increases in BTD patients over time, enzyme activity increase is most prevalent in carriers of *BTD* c.1330G>C variant, repeated biotinidase level measurements are needed to better evaluate BTD, consanguineous marriage may be a risk factor for worse prognosis in BTD, and enzyme activity is most impaired in compound mutation carriers. We are of the opinion that these findings would contribute to the knowledge of BTD management.

## Data Availability

The anonymized data collected are available as open data via the Mendeley Data online data repository: doi: 10.17632/kpk48thdzg.1 (data.mendeley.com/datasets/kpk48thdzg/1).

## Supplementary material

Supplementary Figure 1 Overall variant allele frequencies for the examined genetic mutations in the BTD gene.
