# Supplementary material for: Assessment of biotinidase activity changes over time in biotinidase deficient patients
Source: Front Pediatr. 2026 Mar 2;14:1694031. doi: 10.3389/fped.2026.1694031 (PMC12989555; doi:10.3389/fped.2026.1694031)
Supplement: Supplementary file 1 [file Table1.docx]

**Supplementary Tables**

**Supplementary Table 1.** Used PCR primers for *BTD* gene amplification

| **Region-1** | **Forward** | TTCCTGCCTCCTTTCCTAATCA |
| --- | --- | --- |
|  | **Reverse** | CACACAAGGGATTTTTCCCCAC |
| **Region-2** | **Forward** | GCAGGATTCTTTATTCAGCTG |
|  | **Reverse** | GCAATCTGCTCTGTATGAGAG |
| **Region-3** | **Forward** | CCTGCCATCTGATAACAGAC |
|  | **Reverse** | CTGACTTAGATCACCTCTGTG |
| **Region-4** | **Forward-1** | TCAATCTCCTGACCTCATG |
|  | **Reverse-1** | GGTTCCATTATTGCTGAACACGA |
|  | **Forward-2** | CTTGTCATAGCAGTGACC |
|  | **Reverse-2** | GGTCCGTTTCACCTGTTGCA |
|  | **Forward-3** | ATACACACCCCTCTGGAGTC |
|  | **Reverse-3** | GTAAGTGCCATGTACTGT |
|  | **Forward-4** | AGAAGGATGCTCAGGAAGTC |
|  | **Reverse-4** | CAACATGATGGCCAGAGTC |

**Supplementary Table 2.** Comparisons of genotype and allele frequencies for the *BTD* c.1330G>C and c.470G>A mutations between the groups

| ***BTD* c.1330G>C Mutation** | | | | | | | |
| --- | --- | --- | --- | --- | --- | --- | --- |
|  | **Genotype** | | | | **Allele** | | |
|  | **GG, n (%)** | **GC, n (%)** | **CC, n (%)** | **p Value** | **G, n (%)** | **C, n (%)** | **p Value** |
| **Partial Deficiency** | 95 (56.5) | 50 (29.8) | 23 (13.7) | 0.3 | 240 (71.4) | 96 (28.6) | 0.3 |
| **Normal Activity** | 13 (54.2) | 5 (20.8) | 6 (25) |  | 31 (64.6) | 17 (35.4) |  |
| ***BTD* c.470G>A Mutation** | | | | | | | |
|  | **Genotype** | | | | **Allele** | | |
|  | **GG, n (%)** | **GA, n (%)** | **AA, n (%)** | **p Value** | **G, n (%)** | **A, n (%)** | **p Value** |
| **Partial Deficiency** | 131 (78) | 25 (14.9) | 12 (7.1) | N/A | 287 (85.4) | 49 (14.6) | 0.91 |
| **Normal Activity** | 18 (72) | 7 (28) | 0 (0) |  | 43 (86) | 7 (14) |  |

N/A: not available

**Supplementary Table 3.** Comparison of the homozygous, compound, and single mutation carriers according to BTD diagnosis

|  | **Homozygous Mutation**  **n (%)** | **Compound Mutation**  **n (%)** | **Single Mutation**  **n (%)** | **p Value** |
| --- | --- | --- | --- | --- |
| **Profound Deficiency** | 0 (0) | 2 (100) | 0 (0) |  |
| **Partial Deficiency** | 38 (33) | 57 (49.6) | 20 (17.4) | 0.181 |
| **Normal Activity** | 6 (26.1) | 9 (39.1) | 8 (34.8) |  |

p-value is for the comparison between patients with partial BTD and patients with normal biotinidase activity.
