# Supplementary figures and images for: Assessment of biotinidase activity changes over time in biotinidase deficient patients
Source: Front Pediatr. 2026 Mar 2;14:1694031. doi: 10.3389/fped.2026.1694031 (PMC12989555; doi:10.3389/fped.2026.1694031)

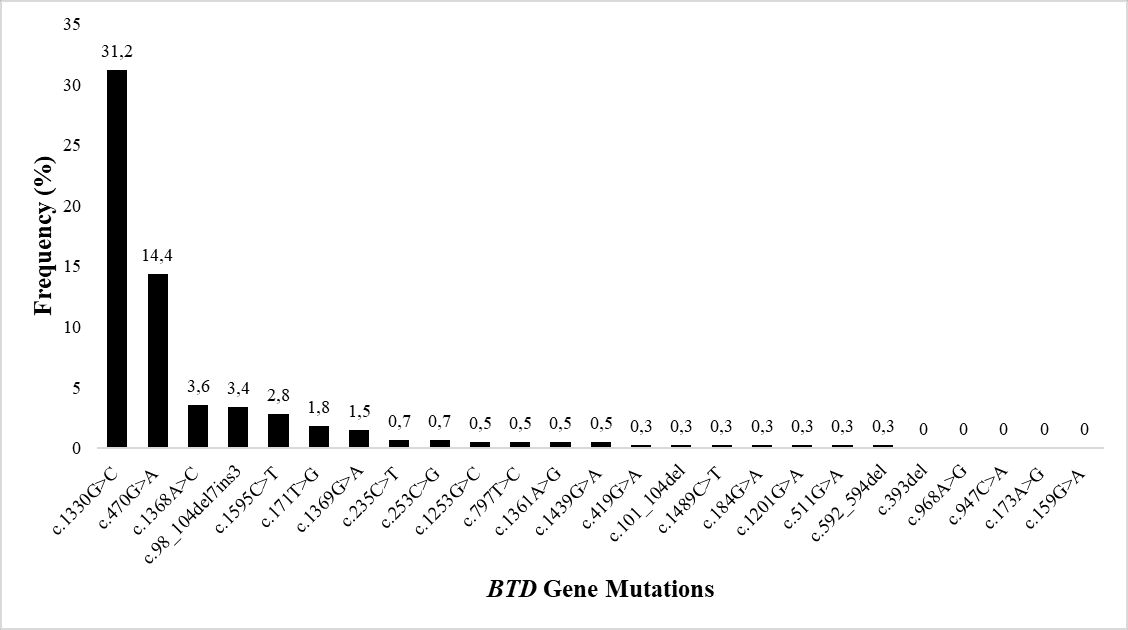

Supplement: Supplementary Figure 1 — Overall variant allele frequencies for the examined genetic mutations in the BTD gene. [file Image1.jpeg]
